# Graphene Oxide–Silver Nanoparticle Nanohybrids: Synthesis, Characterization, and Antimicrobial Properties

**DOI:** 10.3390/nano10020376

**Published:** 2020-02-21

**Authors:** Mónica Cobos, Iker De-La-Pinta, Guillermo Quindós, M. Jesús Fernández, M. Dolores Fernández

**Affiliations:** 1 Department of Polymer Science and Technology, Faculty of Chemistry, University of the Basque Country (UPV/EHU), Paseo Manuel Lardizábal 3, 20018 San Sebastián, Spain; monica.cobos@ehu.es (M.C.); mjesus.fernandez@ehu.es (M.J.F.); 2 Department of Immunology, Microbiology and Parasitology, Faculty of Medicine and Nursing, University of the Basque Country (UPV/EHU), Barrio Sarriena s/n, Leioa 48940, Spain; iker.delapinta@ehu.es (I.D.-L.-P.); guillermo.quindos@ehu.eus (G.Q.)

**Keywords:** graphene oxide, silver nanoparticles, nanohybrids, antimicrobial activity

## Abstract

Drug resistance of pathogenic microorganisms has become a global public health problem, which has prompted the development of new materials with antimicrobial properties. In this context, antimicrobial nanohybrids are an alternative due to their synergistic properties. In this study, we used an environmentally friendly one-step approach to synthesize graphene oxide (GO) decorated with silver nanoparticles (GO–AgNPs). By this process, spherical AgNPs of average size less than 4 nm homogeneously distributed on the surface of the partially reduced GO can be generated in the absence of any stabilizing agent, only with ascorbic acid (L-AA) as a reducing agent and AgNO_3_ as a metal precursor. The size of the AgNPs can be controlled by the AgNO_3_ concentration and temperature. Smaller AgNPs are obtained at lower concentrations of the silver precursor and lower temperatures. The antimicrobial properties of nanohybrids against Gram-negative bacteria *Escherichia coli* and *Pseudomonas aeruginosa*, Gram-positive *Staphylococcus aureus*, and the yeast *Candida albicans* were found to be concentration- and time-dependent. *C. albicans* and *S. aureus* showed the highest susceptibility to GO–AgNPs. These nanohybrids can be used as nanofillers in polymer nanocomposites to develop materials with antimicrobial activity for applications in different areas, and another potential application could be cancer therapeutic agents.

## 1. Introduction

The emergence of nanotechnology has led to the development of nanomaterials, including inorganic nanoparticles with antimicrobial properties, such as silver nanoparticles (AgNPs). These nanoparticles have been proposed as an alternative for mitigating the incidence of healthcare-associated infectious diseases and as a possible solution to the increasing antibiotic resistance problem. Several physical, chemical, and biological methods have been reported to synthesize AgNPs [1,2,3,4,5]. Among chemical processes, chemical reduction, based on the reduction of a silver salt solution by a reducing agent, is the most simple, cost-effective, and frequently applied method. The reduction of silver ions (Ag^+^) results in silver atoms (Ag^0^), which agglomerate into oligomeric clusters leading to the formation of colloidal AgNPs. However, they have low colloidal stability when used in liquid systems, and aggregation to form clusters occurs owing to the high surface area of nanoparticles. Stabilizing agents in the synthesis of AgNPs play a crucial role in the formation of nanoparticles with controlled size and a well-defined shape [6]. Nanoparticle size and shape dictate its physicochemical properties [7,8]. Thus, several studies have reported that the antimicrobial activity of AgNPs is both size and shape dependent [7,9,10]. Graphene oxide (GO), the oxidized form of graphene, is an appropriate material to disperse and stabilize silver since it combines a large specific surface area and abundant oxygenated functional groups. In addition, GO produces stable dispersions in water. The oxygen-containing groups act as anchoring sites for the attachment of metal nanoparticles. GO sheets act as a support for growth and stabilization of AgNPs.

The synthesis of GO–AgNP hybrids by different approaches has been reported, where different reducing agents have been used. Moreover, in most of these studies, a stabilizing agent is used to prevent silver nanoparticle agglomeration and control its structure. Bao et al. used hydroquinone as the reductant and citrate as the stabilizer [11]. Das et al. employed NaBH_4_ in the absence and presence of trisodium citrate as a stabilizing agent [12,13]. Shen et al. used a mixed reducing agent, ethylene glycol and NaBH_4_ [14]. In the study of Chook et al. a microwave approach with glucose as a reducing agent was used [15]. Ma et al. also reported the preparation of Ag–GO composites under ultrasonication using glucose as a reducing agent [16]. Tang et al. [17], Fonseca de Faria et al. [18], and Yuan et al. [19] used sodium citrate as a reducing and stabilizing agent at boiling temperature, 130 °C and 95 °C, respectively. Hydrazine monohydrate was used as a reductant by Cai et al. to prepare polyethyleneimine-modified reduced graphene oxide-AgNP hybrids [20]. KOH was used as a reducing agent at boiling temperature in the work reported by Pasricha et al. [21]. In the study by Hui et al. AgNP–GO composites were fabricated under ultrasonication with ascorbic acid as the reductant [22]. Shen et al. synthesized Ag-GO composites at 160 °C with ascorbic acid as the reductant and an ionic liquid as a dispersing agent [23]. Several of the mentioned methods have the disadvantage of using toxic reducing agents in the synthesis process.

It has been demonstrated that in these nanostructures GO and AgNPs work synergistically to enhance their properties, such as higher antimicrobial and catalytic activities and thermal conductivity. The synergistic properties of these hybrid materials have proven to be useful in a variety of applications (electronics, catalysis, electrochemical biosensing, drug delivery, and antimicrobial agents) [24,25,26,27,28,29]. 

Both graphene and AgNPs have been combined with polymer matrices to develop antimicrobial materials [30]. Polymer nanofibrous patches containing antimicrobial agents have been fabricated via electrospinning for application in various fields [31]. More recently, Alenezi et al. [32] developed a new route that improves the quality and production yield of nanofibers by using the pressurized gyration technique. The microbial properties of graphene nanoplatelet-loaded polymeric fibers obtained by this process have been studied by Matharu et al. [33].

The increasing prevalence of antimicrobial resistance has become one of the major health problems worldwide; thus, the need to develop new therapeutic strategies against multi-drug resistant microorganisms is crucial. Many infectious agents are transmitted by human contact or through objects, food, water, and animals. Therefore, the use of antimicrobial materials in the composition, for instance, of biomedical devices or in food transport containers can help reduce microbial contamination and pathogen transmission [34].

In this study, graphene oxide-silver nanoparticle nanohybrids (GO–AgNPs) were synthesized via a one-step environmentally friendly approach using an aqueous dispersion of GO, AgNO_3_ as a metal precursor, and ascorbic acid as a green reductant, without any stabilizing agent. The physicochemical properties of the GO–AgNP nanohybrids and the influence of metal precursor concentration and temperature on AgNP size were evaluated. In addition, the antimicrobial activity of GO and nanohybrids against Gram-negative bacteria *Escherichia coli* and *Pseudomonas aeruginosa*, Gram-positive bacterium *Staphylococcus aureus,* and the yeast *Candida albicans* was also investigated. The AgNPs had a spherical shape and an average size less than 4 nm. The nanohybrids exhibited species-specific antimicrobial activities. 

## 2. Materials and Methods 

### 2.1. Materials

Graphite flakes were purchased from Alfa Aesar (Karlsruhe, Germany) (99.8%, 325 mesh) and sodium nitrate (NaNO_3_) was obtained from Merck (Darmstadt, Germany). Sulphuric acid (H_2_SO_4_, 98%), potassium permanganate (KMnO_4_), hydrogen peroxide (H_2_O_2_, 30 wt% aqueous solution), hydrochloric acid (HCl, 37% aqueous solution), and ammonium hydroxide were acquired from Panreac (Barcelona, Spain), while L-ascorbic acid (L-AA), silver nitrate (AgNO_3_), phosphate-buffered saline (PBS), and culture medium Roswell Park Memorial Institute (RPMI) were supplied by Sigma–Aldrich (Munich, Germany, and St. Louis, MO, USA). *Escherichia coli* ATCC 25922 (Gram-negative bacteria) and *Staphylococcus aureus* ATCC 25923 (Gram-positive bacteria) were obtained from CECT (Spanish Type Culture Collection, Valencia, Spain), *Candida albicans* SC5314 was obtained from the American Type Culture Collection (ATCC, Manassas, Virginia, WV, USA), and brain heart infusion (BHI) broth and Mueller–Hinton broth were supplied by Condalab (Madrid, Spain). All chemicals were used as received without further purification.

### 2.2. Synthesis of Graphene Oxide (GO) and Graphene Oxide–Silver Nanoparticle Nanohybrids (GO–AgNPs)

To obtain graphene oxide, first, graphite oxide (GrO) was prepared from the oxidation of graphite, which was then exfoliated to obtain GO. GrO was synthesized from natural graphite flakes following a modified Hummers method as described in our previous work [35]. Thus, 6 g of KMnO_4_ was slowly added to a mixture of graphite (2 g), H_2_SO_4_ (50 mL), and NaNO_3_ (1 g) at 0 °C. After 1 h at 35 °C in a water bath, 100 mL de-ionized water was added slowly. Then, 10 mL of 30% H_2_O_2_ was incorporated into the solution and followed by centrifugation. The solid was then washed repeatedly with water, HCl and deionized water, until neutral pH. Finally, the solid was separated by centrifugation and re-dispersed in water to obtain GrO by freeze-drying. The exfoliation of GrO by ultrasonication (Bandeline Sonopuls HD 3200 homogenizer, Berlin, Germany) led to GO. 

GO–AgNP nanohybrids were synthesized via the in situ method, through the simultaneous reduction of the metal precursor and graphene oxide (GO) using L-ascorbic acid as a green reductant. GrO powder (0.5 mg/mL) was dispersed in deionized water using an ultrasonic bath for 1 h to obtain GO, and the pH was adjusted to 10 by the addition of ammonium hydroxide. Then, the desired amount of an aqueous AgNO_3_ solution (to reach 1.5 mM or 2.0 mM) was slowly added to the dispersion under continuous stirring in the absence of light and heated at 60 °C in an oil bath. Next, an aqueous solution of L-AA was added at a concentration that maintained the weight ratio between L-AA and AgNO_3_ fixed at 2.07. The reaction mixture was held at 60 °C for 1 hour and was then cooled, dialyzed, and centrifuged at high speed repeatedly. Finally, GO–AgNP powder was obtained by freeze-drying. The experiments were also carried out at 80 °C to investigate the effect of temperature on the properties of AgNPs. Table 1 lists the prepared nanohybrid sample names and the reaction conditions.

### 2.3. Characterization 

Fourier transform infrared spectroscopy (FTIR), ultraviolet–visible absorption spectroscopy (UV–vis), X-ray photoelectron spectroscopy (XPS), Raman spectroscopy, X-ray diffraction (XRD), and transmission electron microscopy (TEM) were employed to evaluate the structure and the morphology of the samples. 

#### 2.3.1. FTIR Analysis

FTIR spectra of GO and GO–AgNPs were recorded on a Thermo Nicolet iS10 spectrometer (Thermo Fisher Scientific, Madison, WI, USA) equipped with an attenuated total reflectance accessory (ATR). 

#### 2.3.2. UV–vis Analysis

A Perkin Elmer Lambda 25 spectrometer (Shelton, CT, USA) was used to collect the UV–vis absorption spectra between 200 and 800 nm of graphene materials. 

#### 2.3.3. XPS Analysis

The analysis of the surface chemistry of GO and GO–AgNPs was performed by XPS on a SPECS system with a monochromatic Al Kα X-ray source (1486.6 eV) and a Phoibos 150 1D-DLD analyzer (Berlin, Germany). The core level spectra were obtained at a photoelectron take-off angle of 90°, measured with respect to the sample surface. The XPS survey–scan spectra were collected using a pass energy of 80 eV, 1 eV energy step, and 0.1 s dwell time. The individual high-resolution spectra were acquired using a pass energy of 30 eV, 0.1 eV energy step, and 0.1 s dwell time. 

#### 2.3.4. Raman Analysis

Raman spectra of GO and GO–AgNPs were obtained with a Renishaw Invia microscope (Gloucestershire, UK) with a laser frequency of 514 nm as an excitation source. The spectra were measured from 500 to 3500 cm^−1^. 

#### 2.3.5. XRD Analysis

The XRD spectra of graphene materials were recorded using a Malvern Panalytical (Almelo, the Netherlands) X’PERT PRO automatic diffractometer operating at 40 kV and 40 mA in theta–theta configuration, a secondary monochromator with Cu-Kα radiation (λ = 1.5418 Å), and a PIXcel solid state detector (active length in 2*θ* 3.347°). Data were collected from 1 to 50° 2*θ* for GO and from 1 to 90° 2*θ* for GO–AgNP samples (step size = 0.026° and time per step = 80 s, total time 20 min) at room temperature. A variable divergence slit giving a constant 5 mm area of sample illumination was used. The interlayer separation in the graphene material was calculated by Bragg´s law (*λ* = 2d sin*θ*). 

#### 2.3.6. TEM Analysis

TEM images were taken using a Philips Tecnai G2 20 TWIN TEM instrument (Eindhoven, the Netherlands) at 200 kV accelerated voltage. A drop of the diluted GO–AgNP nanohybrid suspensions was placed onto coated cooper grids. 

#### 2.3.7. Thermogravimetric Analysis (TGA)

TGA tests were performed on a TG-Q-500 (TA instruments, New Castle, DE, USA) under nitrogen at a heating rate of 10 °C/min, from 40 to 600 °C.

### 2.4. Microbial Strains and Culture

Three well-described bacterial species and one clinically relevant yeast were evaluated: Gram-negative bacteria *E. coli* ATCC 25922 and *P. aeruginosa* ATCC 27853, the Gram-positive bacterium *S. aureus* ATCC 25923, and *C. albicans* SC5314. Briefly, microorganisms were harvested on plate count agar (PCA) for 24 h at 37 °C from frozen stock, and inoculum was prepared from single colonies grown to stationary phase in BHI broth at 37 °C overnight in an orbital incubator under 100 rpm. Cultures were centrifuged (3000 × g, 10 min) and washed twice in PBS. A cell suspension adjusted to a cell density equivalent to 0.5 McFarland (representing approximately 1–5 × 10^8^ cells/mL) was prepared using sterile saline for bacteria, and *C. albicans* was adjusted at 1 × 10^6^ cells/mL upon counting cells in a hemocytometer. Viable counts were enumerated after overnight incubation at 37 °C in PCA.

### 2.5. Antimicrobial Activity Assays for GO and GO–AgNP Nanohybrids. Determination of the Minimum Inhibitory Concentrations (MICs)

The minimum inhibitory concentration (MIC) is defined as the lowest concentration, recorded in mg/L or μg/mL, of an agent that inhibits the growth of a microorganism. In the present study, the MIC of four microorganisms was determined: *C. albicans* SC5314, *S. aureus* ATCC 25923, *E. coli* ATCC 25922, and *P. aeruginosa* ATCC 27853. The EUCAST broth microdilution method (EUCAST EDef 7.3.1) was used to establish the MICs of GO and GO–AgNPs since it is a reference method for antimicrobial susceptibility testing, and one of its main purposes is to establish the activity of new antimicrobial agents. Stock solutions of graphene derivatives at 200-fold the concentration to be analyzed were prepared in order to maintain the same concentration of culture medium in each well of the microdilution plate. Next, these solutions were diluted 100-fold in the corresponding culture medium, which was previously prepared at 2-fold concentration. The culture medium was RPMI with 2% of glucose (RPMI 2% G) and Mueller–Hinton broth (recommended by EUCAST) for *C. albicans* and bacteria, respectively. Subsequently, 100 μL of each dilution was added to each well; the final test concentration for each compound ranged from 0.25 μg/mL to 128 μg/mL. Then, the microbial suspension was diluted 1:10 for *C. albicans* and 1:100 for bacteria in sterile distilled water, and 100 μL was pipetted into each well, obtaining the final inoculum concentration of 5 ×10^4^ CFU/mL and 5 × 10^5^ CFU/mL, respectively. Finally, the microdilution plates were read using a microdilution plate reader (iMark Microplate Reader, BioRad, Hercules, CA, USA) at a wavelength of 450 nm at 0 h and 24 h at 37 °C. The MIC corresponds to the concentration of the compound that resulted in an absorbance reduction of 50% or greater with respect to the absorbance found in the wells of the growth control.

### 2.6. Microbial Growth Kinetics Assay in the Presence of GO–AgNP Nanohybrids

The four previously mentioned microorganisms were analyzed at the final inoculum density of 5 × 10^4^ CFU/mL and 5 × 10^5^ CFU/mL for yeast and bacteria, respectively. The highest GO concentration in the MIC determination assay (128 μg/mL) was tested along with three concentrations of GO–AgNPs, selected on the basis of the MIC value of each microorganism: 64 μg/mL, 32 μg/mL, and 16 μg/mL for *C. albicans* and 128 μg/mL, 64 μg/mL, and 32 μg/mL for *S. aureus*, *E. coli,* and *P. aeruginosa*. Briefly, a microplate was loaded with culture medium, the GO–AgNP hybrid, and the microorganism as mentioned above. The microorganism in the culture medium was considered the growth control (GC), and the different concentrations of the compounds without microorganisms but with the culture medium, the blank. The microplates were placed in a microplate reader (BioScreen C, Labsystem, Helsinki, Finland) configured to read the absorbance at 430–580 nm every hour for 72 hours at 37 °C. Five wells for each compound concentration were used, and the assay was performed in duplicate.

## 3. Results and Discussion

### 3.1. Characterization

GO and GO–AgNPs were physicochemically characterized, firstly, to confirm their formation, and secondly, because the antimicrobial properties depend on the physicochemical properties such as chemical composition, shape, and size of the AgNPs. 

#### 3.1.1. Structure and Morphology

##### FTIR Analysis

**Figure 1:**
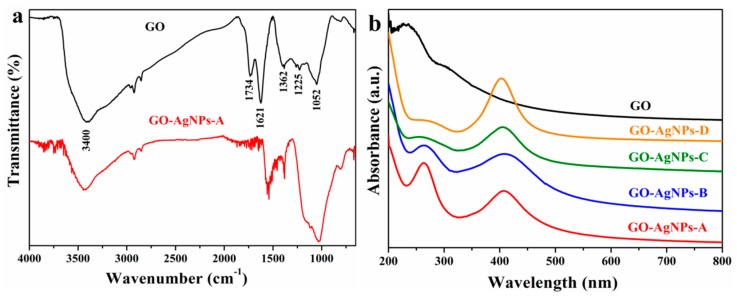
(**a**) FTIR and (**b**) UV–vis spectra of GO and GO–AgNP nanohybrids.

The FTIR spectra of GO and GO–AgNP nanohybrids are displayed in Figure 1a. The GO spectrum shows various bands associated with the vibrational modes of different oxygen-containing functional groups. The band at 3800–3000 cm^−1^ is attributed to the stretching vibrations of structural OH groups and physisorbed water molecules. The bands at 1734 cm^−1^ and 1621 cm^−1^ are related to the C=O carbonyl stretching of COOH groups situated at the edges of the GO sheets and to the deformation vibration of water molecules, respectively. The peaks at 1362 cm^−1^ and 1052 cm^−1^ arise from the bending of tertiary C-OH groups and the vibration of C–O of epoxide groups (C–O–C), respectively. The peak at 1225 cm^−1^ is assigned to C−O−C stretching. For GO–AgNP nanohybrids, the weakening of the carbonyl and hydroxyl bands is observed, as well as the elimination of the band at 1621 cm^−1^. These results indicate the simultaneous partial reduction of GO.

##### UV-Vis Analysis

The UV–vis spectroscopy was used to confirm the formation of GO–AgNP nanohybrids. The UV–vis spectra of GO and GO–AgNP nanohybrids are shown in Figure 1b. For GO, the maximum absorption peak at 230 nm is ascribed to the electronic *π* → *π* * transitions of aromatic C-C bonds, and the shoulder at ~300 nm corresponds to *n* → *π* * transitions of C=O bonds. The absorption spectrum of GO–AgNP hybrid exhibits the red shift of the maximum peak of GO, from 230 to 265 nm, suggesting the simultaneous partly reduction of GO during the preparation of the GO–AgNP nanohybrids and therefore the recovery of the electronic conjugation of the graphene sheets. In addition, the spectrum shows the characteristic surface plasmon resonance (SPR) band of AgNPs (~ 404 nm), which confirms the presence of small spherical-shaped AgNPs on GO [36]. UV-Vis spectroscopy was also used to analyze the effect of reaction temperature and the AgNP precursor concentration on the synthesis of GO–AgNP nanohybrids. As can be seen from Figure 1b, the intensity and shape of the SPR absorption band remain similar at both temperatures when 1.50 mM AgNO_3_ was used. However, the absorbance peak becomes more intense as temperature increases in the case of 2.00 mM AgNO_3_, which can be due to an increase of the number of nanoparticles formed. The position of the SPR band remains practically unchanged as the temperature and silver precursor concentration increase.

##### XPS Analysis

**Figure 2:**
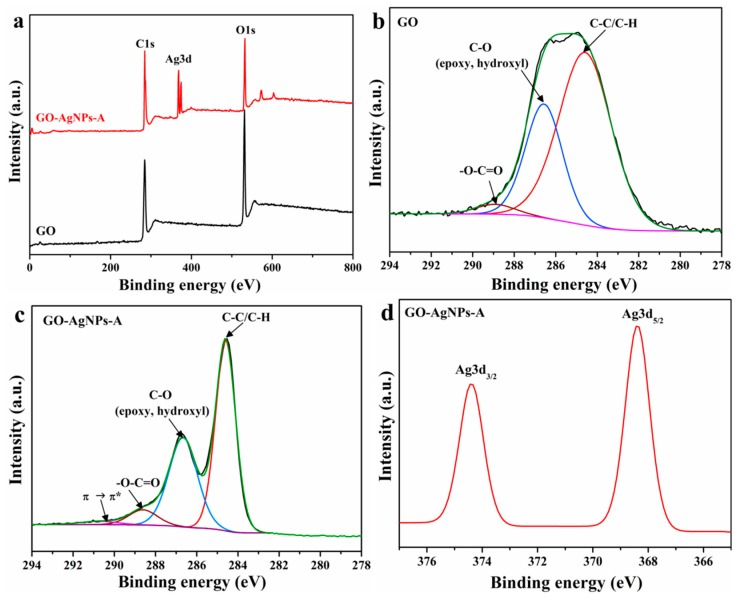
XPS characterization of GO and GO–AgNP-A. **(a)** XPS survey spectra of GO and GO–AgNP-A. **(b)** High-resolution C1s XPS spectrum of GO. **(c)** High-resolution C1s XPS spectrum of GO–AgNP-A. **(d)** High-resolution XPS spectrum of Ag3d of GO–AgNP-A.

XPS characterization was performed to prove both the reduction of GO and the formation of AgNPs. XPS survey spectra of GO and the GO–AgNP-A nanohybrid are shown in Figure 2a. For both samples, the peaks observed at 284.4 eV and 531.4 eV are associated with the peaks of C1s and O1s, respectively. The new peaks at about 368 eV and 374 eV that appear in the spectra of GO–AgNP-A belong to Ag3d. The high-resolution C1s XPS spectra for GO and GO–AgNP-A are displayed in Figure 2b,c. By comparing the two spectra, it can be observed that there is a remarkable reduction in C-O peak intensities, a narrowing of the C-C signal, and the appearance of the shake-up satellite at about 290.5 eV, characteristics of the aromatic C-C. These results suggest the partial reduction of GO during the synthesis of the GO–AgNP nanohybrids. The Ag3d spectrum for the GO–AgNP nanohybrid shows the peaks of Ag3d5/2 and Ag3d3/2 at 368.4 and 374.4 eV, respectively (Figure 2d), indicating that Ag exists in the metallic form.

##### Raman Analysis

**Figure 3:**
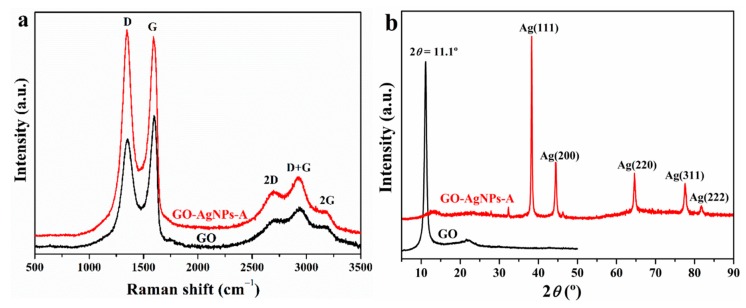
(**a**) Raman spectra and (**b**) XRD patterns of GO and the GO–AgNP nanohybrid.

GO and GO–AgNP nanohybrids were further characterized by Raman spectroscopy. Figure 3a shows the Raman spectra of GO and the GO–AgNP-A nanohybrid. 

The spectra of both samples displayed two characteristic bands, the G band (1598 cm^−1^) that represents the E2g mode within aromatic carbon rings, and the D band (1353 cm^−1^) due to the in-plane carbon ring breathing mode, which is active when defects are present. In addition, several bands were present in the second region from 2250 to 3400 cm^−1^. The Raman data are listed in Table 2. The attachment of AgNPs to the GO surface led to the shift of both bands to lower wavenumbers, to a more intense D band, and to an increase in the I_D_/I_G_ intensity ratio, indicating the formation of more sp^2^ domains with smaller average size as a consequence of the partial reduction of GO. In addition, an increase in the Raman scattering was observed and is ascribed to the surface-enhanced Raman scattering (SERS) effect of Ag nanoparticles that results from the intense local electromagnetic fields of Ag nanoparticles [37].

##### XRD Analysis

The crystalline nature of the synthesized GO and GO–AgNP nanohybrid was analyzed by XRD (Figure 3b). The pattern of GO displays the (001) diffraction peak at 2*θ* = 11.1°; according to the Bragg diffraction formula, the *d* spacing of GO is 0.79 nm. Regarding GO–AgNPs, the X-ray diffraction patterns shows strong Bragg reflections at 38.3°, 44.2°, 64.5°, 77.3°, and 81.7º of 2*θ*, which correspond to the (111), (200), (220), (311), and (222) crystal planes, respectively, of the face-centred cubic crystal structure of AgNPs. These results corroborate the formation of AgNPs on the GO surface. In addition, the non-appearance of the GO diffraction peak after the attachment of AgNPs onto its surface suggests the exfoliation of GO–AgNP sheets.

##### TEM

**Figure 4:**
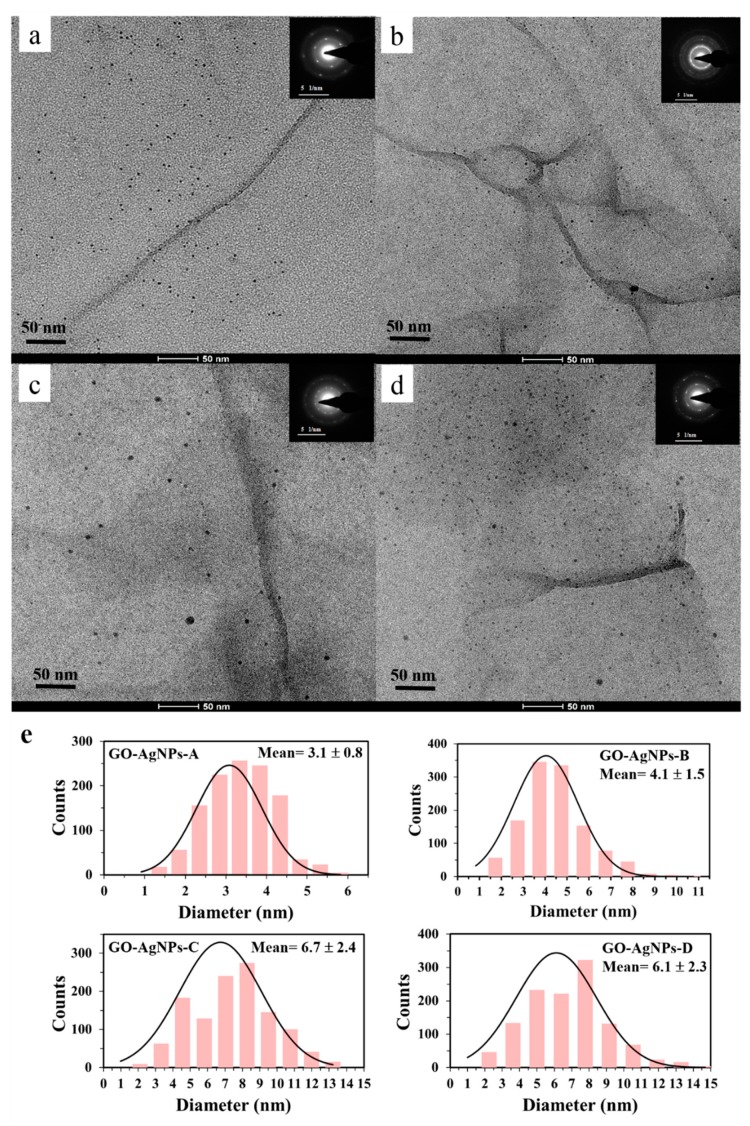
TEM images of the GO–AgNP nanohybrids, where the insets in (a-d) present the SAED of the nanoparticles (rings and spots). (**a**) GO–AgNP-A; (**b**) GO–AgNP-B; (**c**) GO–AgNP-C; (**d**) GO–AgNP-D; (**e**) particle size distributions of AgNPs.

The morphology of the synthesized GO–AgNP nanohybrids was investigated by TEM. Figure 4 shows TEM images, selected area electron diffraction (SAED) patterns of GO–AgNP nanohybrids, and particle size distributions of AgNPs on GO sheets. The micrographs confirm the decoration of GO with AgNPs. For all samples, spherical AgNPs well dispersed throughout the GO surface were observed. For each sample, 1200 nanoparticles in several GO–AgNP micrographs were analyzed to determine the AgNP sizes. 

It can be seen that the size of the AgNPs is dependent on the AgNO_3_ concentration and temperature. The results indicate that the smallest AgNPs are formed at the lowest silver precursor concentration and temperature. Under these conditions, 1.50 mM AgNO_3_ and 60 °C, the reaction leads to silver nanoparticles whose size ranges from 0.9 nm to 5.8 nm, with an average size of 3.1 ± 0.8 nm (Figure 4a,e), where about 90% of the AgNPs counted are under 4 nm in diameter. An increase in the silver precursor concentration results in an increase of the particle size and a broader size distribution when the reaction temperature is 60 °C (Figure 4b,e). Thus, for the GO–AgNP-B nanohybrid, the particle size ranges from 0.7 nm to 10.8 nm, with an average size of 4.1±1.5 nm, where only 51% of nanoparticles exhibit diameters less than 4 nm. These findings are in accordance with previous results reported by other authors [12,22,38,39,40]. On the other hand, in Figure 4c,d,e, it can be seen that both the size and size distribution are unaffected by the AgNO_3_ concentration when the reaction temperature is maintained at 80 °C. Thus, the effect of AgNO_3_ concentration on AgNP size and size distribution is significant when the synthesis is performed at lower temperature.

The effect of temperature on silver nanoparticles size can be seen in Figure 4e. It may be noted that as the temperature increases to 80 °C, the nanoparticle size increases and the size distribution becomes broader. Similar results have been reported in the literature [41,42]. The nucleation of AgNPs on the graphene oxide surface has been explained through the interaction of silver cations with the oxygen functional groups. The negatively charged GO sheets allow the attachment of positively charged metal ions via electrostatic interactions. The addition of L-AA reduces both the Ag^+^ species to AgNPs and GO to reduced GO. At higher temperature, the reduction rate of the silver precursor increases, and the nanoparticle growth reaction rate is faster. From the above results, it can be inferred that both the silver precursor concentration and temperature are the key parameters for controlling the size of AgNPs. The sizes of the nanoparticles synthesized in this work are smaller than those reported in the literature using the solution phase chemical reduction method, either with or without stabilizing agent. In the present work, only the silver precursor and the reducing agent were used to prepare the nanohybrids, whereas in most of the reported studies, a stabilizing agent, ultrasonication or a dispersing agent were also employed.

Several studies have reported the synthesis of AgNPs supported on graphene oxide by using the solution phase chemical reduction method. Pasricha et al. reported the synthesis of Ag-GO nanocomposites by chemical reduction of a silver sulfate precursor by GO in the presence of aqueous KOH at boiling temperature [21]. The results revealed the formation of silver nanoparticles with sizes in the range of 3−12 nm. Shen et al. synthesized Ag-chemically converted graphene (CCG) by an in situ solution-based chemical approach with mixed reducing agents at 110 °C [14]. The AgNPs on CCG sheets had a size in the range of 5−10 nm. Das et al. reported the preparation of AgNPs using AgNO_3_ with sodium borohydride in the presence of GO [12]. The size of the AgNPs obtained by this method was in the range of 5–25 nm. Tang et al. prepared GO–Ag nanocomposites with different Ag/GO ratios by chemical reduction of the AgNO_3_ precursor with sodium citrate at boiling temperature [17]. The AgNPs that attached onto the GO sheet surface were found to have an average diameter of about 46 nm and 68 nm depending on the Ag/GO ratio. Das et al. reported the synthesis of AgNPs on GO sheets by chemical reduction of silver metal ions by sodium borohydride in the presence of trisodium citrate as a stabilizing agent, which showed the formation of silver nanoparticles with a particle size of 2–25 nm [13]. The results reported by Fonseca de Faria et al. showed GO sheets decorated with 7.5 nm-sized Ag nanoparticles [18]. In their study the GO–Ag nanocomposite was prepared in the presence of silver nitrate and sodium citrate at 130 °C. Hui et al. reported the formation of AgNPs with an average size ranging from 15 to 55 nm, depending on the ultrasonication time of the mixture of AgNO_3_, GO and vitamin C [22]. 

The insets in Figure 4a–d present the SAED patterns of synthesized AgNPs on GO sheets. They exhibit diffraction rings and bright spots ascribed to face-centred cubic (fcc) metallic silver. The rings correspond to the crystallographic planes (111), (200), (220), and (311) of AgNPs. These results are in agreement with those of XRD and indicate the polycrystalline nature of silver.

#### 3.1.2. Thermal Stability

**Figure 5:**
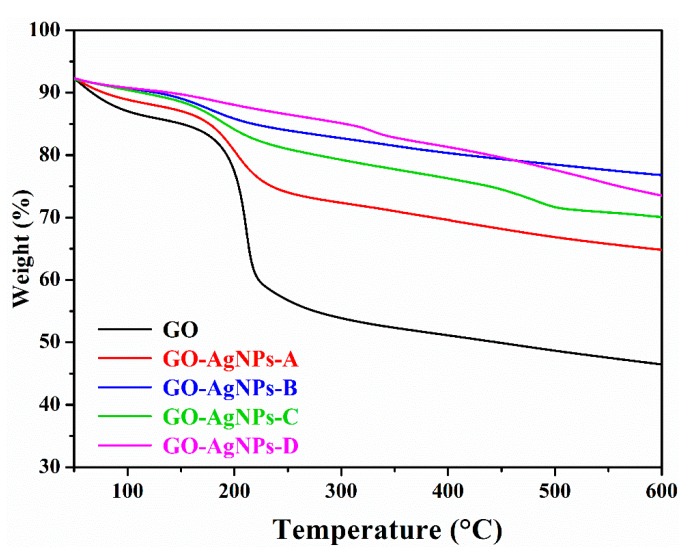
TGA curves of GO and GO–AgNP nanohybrids.

The thermal stability of GO and GO–AgNP nanohybrids was measured by TGA under a nitrogen atmosphere. For GO and all GO–AgNP nanohybrids, the TGA mass loss curves display two decomposition steps (Figure 5). The first mass loss, attributed to the elimination of interlaminar water, is found at 50−120 °C, and the second, where the decomposition of the oxygen groups occurs, at 140–300 °C. During this step, the largest mass loss corresponds to GO (~32%), and decreases as the concentration of AgNO_3_ and temperature increase, being 16.1, 7.7, 12.2, and 5.4% for GO–AgNP-A, GO–AgNP-B, GO–AgNP-C, and GO–AgNP-D, respectively. From these results, it can be concluded that the most stable hybrid is the one obtained at the highest silver precursor concentration and the highest temperature. The improvement in the thermal stability is attributed to the deoxygenation and better graphitization of the hybrids because of the partial reduction of GO.

#### 3.1.3. Antimicrobial Activity Assessment

Healthcare-associated infectious diseases are a relevant medical problem because they prolong hospital stay and cause long-term disability and additional costs for health systems, patients and their families, and preventable deaths. Moreover, treatment of these diseases increases the possibility of selecting multi-drug resistant microorganisms. The most frequent etiological agents are Gram-negative (*E. coli* and *P. aeruginosa*) and Gram-positive (*S. aureus*) bacteria and yeasts (*C. albicans*) [43,44]. These four microorganisms were selected in order to understand the behavior of GO–AgNP nanohybrids on different microbial structures such as Gram-positive and Gram-negative bacteria as well as yeasts. These infectious agents are transmitted mainly by contact with people who carry or are infected by them, as well as through objects (fomites), food, water, and animals. All four microorganisms can become part of the human microbiota and can subsequently cause opportunistic infections. Epidemic strains of these species have also been described that cause moderate and severe infections in people without any underlying disease or immunodeficiency [45]. These species are among the most frequent and important causes of healthcare-associated infectious diseases. They are also problematic species due to resistance or multi-resistance to antibiotics. Gram-positive *S. aureus* is a major human pathogen that causes a wide range of clinical infections. It is a leading cause of bacteremia and one of the most important causes of death associated with bloodstream infection [46]. Antibiotic resistance is common, with the most problematic strains being those called MRSA (methicillin-resistant *S. aureus*). Gram-negative *E. coli* is a major cause of bloodstream infection, as well as the most common pathogen causing urinary tract infection and catheter-associated urinary tract infections, and a critical antimicrobial resistance issue [47]. Multidrug resistance is common in *E. coli*: Among the most frequent mechanisms are the production of beta-lactamases and modifications of antibiotic targets. Gram-negative *P. aeruginosa* is the main cause of ventilated-associated pneumonia in the intensive care unit and a common cause of other nosocomial infections exhibiting innate antibiotic multi-drug resistance [48]. We also included *C. albicans* because it is the most important cause of healthcare-associated fungal diseases (mycoses). It is also the most frequent cause of superficial and deep mycoses. In addition, antifungal resistance is reported in this species although not having the frequency and relevance of resistance in *Staphylococcus*, *Escherichia* or *Pseudomonas*. *C. albicans* is the most relevant fungal pathogen since *Candida* species are ranked as the fourth or fifth most common nosocomial bloodstream pathogen in the USA and some European countries, with mortality rates as high as 45% [44,49].

The antimicrobial activity of GO and GO–AgNP nanohybrids was analyzed by means of the MIC determination and the microbial growth kinetics assay of *E. coli* ATCC 25922, *P. aeruginosa* ATCC 27853, *S. aureus* ATCC 25923, and *C. albicans* SC5314. The strains selected in the current study are recommended for in vitro susceptibility testing. The GO–AgNP-A sample was selected for the study of antimicrobial properties. MIC values are shown in Table 3.

For the GO sample with concentrations within the range of 0.25 μg/mL to 128 μg/mL, a 50% reduction in absorbance with respect to the absorbance of the growth control of any of the four microorganisms studied was not observed. Therefore, the GO used in the present study did not exhibit antimicrobial activity at the concentrations up to maximum tested 128 μg/mL. In the case of GO–AgNP-A, the growth of the microorganisms was inhibited by at least 50% above a certain concentration, which depends on the microorganism. The MIC value for Gram-negative bacteria was 64 μg/mL, while for *S. aureus* and *C. albicans,* 32 μg/mL, after 24 h exposure. 

**Figure 6:**
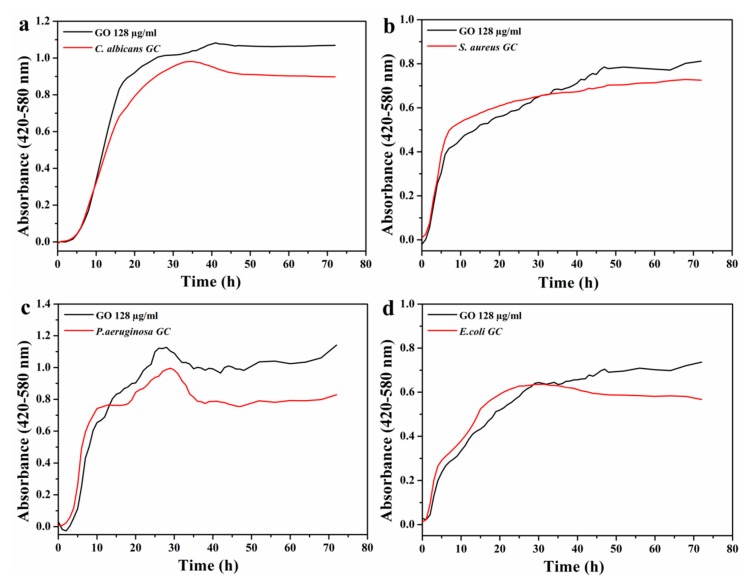
Microbial growth kinetics in contact with GO. (**a**) *C. albicans*, (**b**) *S. aureus*, (**c**) *P. aeruginosa,* and (**d**) *E. coli*.

The microbial growth kinetics test offers an interesting approach to study the antimicrobial activity since it takes into consideration the influence of two important variables: the amount of nanomaterial and the exposure time (longer than in the MIC test) of microbial cells to the nanomaterial. The absorbance of the microplate wells was monitored every hour, which allowed us to establish the growth curves (Figure 6). Owing to the lack of antimicrobial activity found for GO in the previous assay, it was used as a negative inhibition control in this test, and the growth kinetics of the microorganisms were observed in the presence of the highest GO concentration previously tested (128 μg/mL) for 72 h. It can be noted that GO did not prevent the growth of any of the microorganisms used in the present study, confirming once again the lack of antimicrobial activity in the concentration range studied. In addition, GO acts as a growth stimulator, as the growth curve of microorganisms in contact with the nanomaterial exceeded the absorbance of those that had not been in contact, which is in accordance with the results reported in the literature [50,51]. 

Several studies have investigated the antimicrobial properties of GO, and the reported results have been controversial [50,51,52,53]. The discrepancies in the published data have been attributed to differences in the physical-chemical characteristics of the GO used. The size, surface area, functional groups, oxygen content, surface roughness, layer number, purity, and arrangement mode of GO sheets affect their antimicrobial properties. Both the graphite source and the level of oxidation/exfoliation, governed by the preparation method, determine most of these features. The lateral size of GO sheets has been shown to affect their antimicrobial activity. Higher antibacterial activity is displayed by small lateral-sized GO when in the form of surface coating [54], whereas large sheets are more toxic to bacteria for dispersed GO [55]. In addition to the above-mentioned factors, which depend to a very high extent on the type of graphite, the antimicrobial effects of GO are influenced by its concentration, the incubation conditions, exposure time, and the characteristics of microorganisms used. Liu et al. [52] demonstrated that several graphene material dispersions had concentration- and time-dependent antibacterial activity. In their study, the incubation of GO dispersions (at concentrations from 10 to 80 μg/mL) with *E. coli* cells showed the highest antibacterial activity among the different graphene-base materials studied and an almost complete loss of cell viability at a concentration of 80 μg/mL. Moreover, most of the bacterial inactivation was observed in the first hour of incubation. Hu et al. reported the growth inhibition of *E. coli* bacteria by GO, being almost completely suppressed when treated with 85 μg/mL GO for 2 h [53]. Other studies, however, have shown the contrary. In the study by Nguyen et al., the ineffectiveness of GO (up to 400 μg/mL) against *E. coli* was demonstrated [56]. The lack of antifungal activity against *C. albicans* by GO was demonstrated in the studies carried out by Li et al. [57] and Al-Thani et al. [58]. Ruiz et al. reported a rapid and irreversible attachment of bacterial cells to GO [50].

Physical and chemical interactions between GO and bacterial cells are responsible for the antibacterial properties of the GO. When bacterial cells come into direct contact with the sharp edges or basal planes of GO, membrane stress of bacteria can be induced, resulting in disruption and physical damage to cell membranes. The sharp edges of graphene sheets act as nanoknives, cutting the bacterial cell membrane, which leads to the leakage of intracellular components followed by cell death [59]. Wrapping and trapping of bacterial membranes by the flexible thin sheets of GO after their direct contact has also been proposed as another antimicrobial mechanism of the graphene materials [54,55,60]. In this scenario, bacterial cells die by being isolated from the growth medium. Oxidative stress is considered a predominant mechanism of bacterial inactivation of GO [59,61]. In bacterial cells exposed to GO, oxidative interactions play a key role due to the oxidation capacity of graphene material. Oxidative stress can occur through either a reactive oxygen species (ROS)-dependent or a ROS-independent pathway. In the first case, oxidative stress is mediated by the production of ROS, which can damage cellular components. In the second one, however, the production of ROS is not involved, being the charge transfer from the cellular membrane to graphene surface that induces cell death. Ruiz et al. [50] investigated the effect of colloidal GO on bacterial (*E. coli*) growth in Luria–Bertani (LB) nutrient broth, observing a dramatic increase in microbial cell proliferation. The microbial assays showed the precipitation of GO in the culture media when bacteria were present. The scanning electron microscopy (SEM) analysis of this precipitate revealed that it was formed by a thick bacterial biofilm containing a large mass of aggregated cells and extracellular polymeric material. The authors suggested that the GO precipitates acted as scaffolds for cell surface attachment, proliferation, and biofilm formation. Chen et al. [51] also found the formation of anaerobic membrane scaffolds by GO suspensions in bacterial growth medium, which facilitated proliferation of gut microbiota. Hui et al. [62] studied the antimicrobial properties of GO in saline and LB broth. The results showed that LB broth made GO inactive, observing an increase in bacterial growth. The loss of antibacterial activity was attributed to the noncovalent adsorption of LB components on GO basal planes. From the above-mentioned studies, it has been inferred that the culture medium plays a key role in the toxicity of GO against bacteria. More recently, Gusev et al. [63], in their study of the interaction of *E. coli* with reduced graphene oxide (rGO), also demonstrated the important role of the culture medium in the antimicrobial properties of rGO.

**Figure 7:**
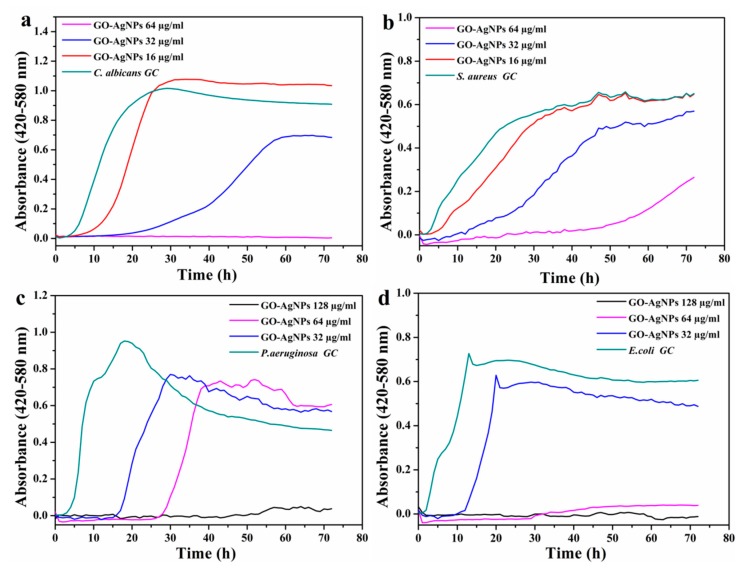
Microbial growth kinetics in contact with GO–AgNPS-A. (**a**) *C. albicans*, (**b**) *S. aureus*, (**c**) *P. aeruginosa,* and (**d**) *E. coli*.

Regarding the GO–AgNP-A sample, MIC/2, MIC, and MICx2 were the nanomaterial concentrations analyzed for each microorganism. The results obtained are presented in Figure 7, where it can be observed that microbial growth was completely prevented at one of the concentrations for all the microorganisms. The curves show four distinct growth phases, lag, log, stationary, and death phases. The lag phase corresponds to the delay before exponential growth begins. In the log or exponential phase, cell division proceeds at a constant rate, whereas in the stationary phase, the conditions become unfavorable for growth and microbes stop replicating and reach an equilibrium level. Finally, in the death phase, cells lose viability. The length of the lag phase is the time that it takes for the different inoculums before an increase in cell number is observed. When microorganisms are cultivated in fresh medium and have to face environmental changes, they enter the lag phase, during which cell growth stops. This allows the cells to adapt to the new situation and to synthesize the cellular components necessary for growth. Depending on the cell structure, the type of antimicrobial agent and its concentration, the growth profile differs [64].

According to Table 3, the MIC value for *C. albicans* was 32 μg/mL, and this can also be confirmed in the growth curves (Figure 7a) where the yeast growth was inhibited for 24 h at this concentration. However, 64 μg/mL of GO–AgNP-A was required to fully inhibit the growth of *C. albicans* after 50 h of incubation with the nanomaterial. For *S. aureus*, the concentration causing at least 50% growth inhibition was also 32 μg/mL, although after 35 h, the inhibitory effect disappeared (Figure 7b), and 64 μg/mL of GO–AgNP-A inhibited the growth of *S. aureus* until 60 h. On the other hand, the two Gram-negative bacteria had an MIC value of 64 μg/mL. However, the antibacterial effect of this concentration on *P. aeruginosa* ceased after 32 h, while with respect to *E. coli,* the antibacterial effect was stable over time (Figure 7c,d). In view of all the results, it could be concluded that the GO–AgNP-A nanohybrid exhibited dose- and time-dependent antimicrobial activity. The concentration of GO–AgNP-A that would hinder the growth of any of these microorganisms, at least for 72 hours, would be higher than 64 μg/mL. These results indicate that the highest susceptibility to the GO–AgNP-A nanohybrid was shown by *C. albicans* and *S. aureus*. The differences found in the antimicrobial effect of the nanomaterial could be due to the different cell wall structures of the four microorganisms, involving a species-specific mechanism as indicated by Tang et al. [17]. 

Several authors have studied the antimicrobial behavior of GO–AgNPs. Fonseca de Faria et al. reported that graphene oxide dispersion lacked antibacterial activity against *P. aeruginosa* at the evaluated concentrations in the study (0.1 to 5.0 μg/mL) after different exposure times, whereas 100% of *P. aeruginosa* cells were fully inhibited after contact with GO/Ag concentrations of 2.5 and 5.0 μg/mL for 30–60 min [18]. Cui et al. investigated the inhibitory effect against *C. albicans* of both GO and the GO–Ag composite [65]. They found that GO did not exhibit growth inhibition of fungal cells, while the study confirmed that GO–Ag had antifungal properties. Moreover, the antifungal property was enhanced when compared with that of bare AgNPs. Jaworski et al. studied the antimicrobial activity of GO-, AgNP-, and GO–AgNP-coated polyurethane foils against *E. coli*, *S. aureus*, *S. epidermidis*, and *C. albicans* at 37 °C for 24 h [66]. The foil coated with GO–Ag showed the strongest antibacterial effect against all tested microorganisms; the growth of bacterial cells was greatly inhibited, while the GO- and AgNP-coated films only slightly reduced it. The yeast *C. albicans* was the most resistant to the deleterious effect of GO–Ag, followed by Gram-positive and Gram-negative bacteria. The study carried out by Tang et al. revealed that both pure GO and AgNPs and the simple mixture of both had no effect on *E. coli* and *S. aureus* at the concentrations studied, whereas the nanocomposite GO–Ag showed dose- and Ag:GO ratio-dependent antibacterial activity [17]. In addition, the results showed that the antibacterial effect of GO–AgNPs was species-specific dependent. The enhanced antimicrobial activity of GO–AgNPs compared to GO and AgNPs is attributed to a synergistic effect of GO an AgNPs and not to an additive effect of both components. Shao et al. investigated the antibacterial activity of a GO–Ag nanocomposite against *E. coli* and *S. aureus* [67]. The synthesized GO–Ag showed a dose-dependent antimicrobial effect and was stronger towards *E. coli* than towards *S. aureus*. Das et al. investigated the antibacterial activity of Ag-GO against *E. coli* and *P. aeruginosa* [12]. They found that *P. aeruginosa* was more sensitive than *E. coli* to Ag-GO. The investigation of the antibacterial activity of a GO–Ag hybrid by Mohammadnejad et al. showed a higher toxicity against *E. coli* than against *S. aureus* [68]. 

The results of the present work suggest that AgNPs play a pivotal role in the antimicrobial activity of GO/AgNP hybrids. The activity of AgNPs is dependent on several parameters, including those inherent to them such as size and shape. The size dependency has been examined in various studies [7,9,10,69,70,71,72]. The smaller the size, the higher the toxicity. Small-sized AgNPs have a larger surface area, resulting in more efficient cell–particle contact. Bare-silver nanoparticles are not stable in aqueous suspensions and have a tendency to aggregate, which limits their applications. GO plays an active role in the enhancement of the stability of the AgNPs, acting as a platform to prevent their agglomeration. The formation mechanism of the GO–AgNP hybrids seems to be through electrostatic interactions between the negatively charged oxygen-containing functional groups on the GO surface and the free silver ions, which are then reduced by the reducing agent, leading to the formation of AgNPs attached to the GO surface [11,18,21,22,67,73,74].

AgNPs have been widely investigated by numerous researchers [10,71,75,76,77,78,79,80,81,82,83], and most of the studies reported have been focused on their antimicrobial properties. Their mechanism of antimicrobial action, however, remains unclear and is still under debate. The possible mechanism includes the attachment of AgNPs to the microbial surface, which is thought to be mediated by electrostatic interaction between the negatively charged cell membrane of many microbes and positive surface charged nanoparticles. Once the nanoparticles have adhered to the microbial cells, they are able to penetrate into them, resulting in damage of their internal components. The cellular internalisation of AgNPs can also generate ROS and induce oxidative stress in bacterial cells due to released Ag^+^ ions. In the presence of dissolved oxygen (in aqueous solutions), the surface of silver nanoparticles is oxidized, and the AgNP oxidative dissolution leads to Ag^+^ ions. Both AgNPs and Ag^+^ ions damage microbial cells by interacting with sulfur-containing proteins (the thiol group of proteins, which are responsible for enzymatic activity) present in microbial membranes or inside these cells, and with phosphorus-containing compounds such as DNA. This chemical interaction can result in the inactivation of such proteins in the microbial cell wall. All these processes will finally lead to the death of microorganisms. The antimicrobial effect of GO–AgNP nanohybrids could be explained by the combined action of direct contact between the AgNPs and the microbial cells and the dissolution of Ag^+^ ions from the silver nanoparticles.

There is an increasing number of patients carrying biomedical devices. Severe infectious diseases associated with their use cause great morbidity and mortality, especially in critically ill people [84]. These types of GO–AgNP nanohybrids have found applications in different areas, as was previously mentioned. Their antimicrobial properties make them appropriate for being used in the field of biomedicine, as well as in the preparation of polymer nanocomposites with antimicrobial properties. In previous work, we incorporated this new class of antimicrobial materials into poly(vinyl alcohol), a biocompatible and biodegradable polymer, to fabricate an antimicrobial polymer nanocomposite [85] that could be a potential wound-dressing material.

The matter of cytotoxicity of nanomaterials is a particularly important aspect to consider in the applications of these materials in the biomedical field. The cytotoxicity of nanomaterials is governed by their physicochemical properties such as size, shape, surface charge, coating, and concentration [34,86]. AgNPs have been found to be toxic to several human cell lines, and their cytotoxicity occurs in a dose-, size-, and time-responsive manner (especially for those with sizes ≤10 nm) by creating ROS, oxidative stress, and DNA damage [87,88,89]. The smaller silver nanoparticles showed higher biological activity in comparison with the larger ones. However, the toxicity threshold for the same cell line was higher for small particles than for large ones. [90]. No cytotoxicity has been observed when AgNPs are coated with appropriate polymers at certain concentrations [91]. From the reported studies, it is inferred that AgNPs have many beneficial effects and applications when the doses are reasonable without adverse effects on human cells [92,93]. Concerning GO–AgNP cytotoxicity, it has been reported that this also occurs in a dose-dependent manner and is affected by the mass ratio of GO:Ag. It has been found that GO–AgNPs are more toxic than their pristine counterparts. The reported studies revealed that the cytotoxicity of GO–AgNPs towards human cell is related to the synergistic effect between GO and AgNPs [87]. As with any other compound, the dose of GO–AgNP nanohybrids to be used in their applications will have to be considered to avoid adverse effects. On the other hand, the anticancer activity of GO, AgNPs, and GO–AgNP nanohybrids against different cancer cells has been reported [94,95]. Silver nanoparticle-decorated graphene oxide shows enhanced anticancer activity compared to GO [96]. Therefore, GO–AgNP nanohybrids could have potential applications in theranostics for cancer [97,98].

## 4. Conclusions

GO–AgNP nanohybrids were successfully synthesized by an environmentally friendly one-step approach in the absence of any stabilizer. The simultaneous reduction of AgNO_3_ and GO in the presence of ascorbic acid resulted in the decoration of partially reduced graphene oxide with uniformly distributed AgNPs of an average size less than 4 nm. The size of the silver nanoparticles was determined by the concentration of the silver precursor and temperature. The effect of the former was more significant at lower temperature. The lower the concentration of silver precursor and the lower the temperature, the smaller the size of the silver nanoparticles anchored on the GO surface. GO dispersion lacked antimicrobial activity against four common pathogens, *E*. *coli*, *P. aeruginosa*, *S*. *aureus,* and *C*. *albicans* over the concentration range investigated, while the nanohybrids exhibited species-specific antimicrobial activity. GO–AgNPs displayed the highest activity against *C. albicans* and *S. aureus*. GO–AgNP nanohybrids induced a dose- and time-dependent toxicity against the four microorganisms. This class of nanohybrids can be used as antimicrobial fillers for the preparation of polymer nanocomposites with antimicrobial properties, which can find applications in different fields, and they could also be considered non-toxic agents for application in cancer therapy. 

## Figures and Tables

**Table 1:** Nomenclature and reaction conditions for graphene oxide (GO) decorated with silver nanoparticle (GO–AgNP) nanohybrids.

Sample	Experimental Conditions	
	Temperature (°C)	Concentration of AgNO_3_ (mM)
GO–AgNP-A	60	1.50
GO–AgNP-B	60	2.00
GO–AgNP-C	80	1.50
GO–AgNP-D	80	2.00

**Table 2:** Raman shift positions and intensity ratio (I_D_/I_G_) of GO and GO–AgNPs.

Material	D	G	2D	D+G	2G	I_D_/I_G_
	(cm^−1^)	(cm^−1^)	(cm^−1^)	(cm^−1^)	(cm^−1^)	
GO	1353	1598	2741	2944	3181	0.83±0.02
GO–AgNPs-A	1345	1594	2712	2934	3176	1.03±0.01

**Table 3:** Minimum inhibition concentrations of GO and GO–AgNP-A.

	MIC (μg/mL)	
Microorganism	GO	GO–AgNP-A
*Escherichia coli*	>128	64
*Pseudomonas aeruginosa*	>128	64
*Staphylococcus aureus*	>128	32
*Candida albicans*	>128	32
